# Importance of genetic parameters and uncertainty of MANIHOT, a new mechanistic cassava simulation model

**DOI:** 10.1016/j.eja.2020.126031

**Published:** 2020-04

**Authors:** Leidy Patricia Moreno-Cadena, Gerrit Hoogenboom, Myles James Fisher, Julian Ramirez-Villegas, Steven Dean Prager, Luis Augusto Becerra Lopez-Lavalle, Pieter Pypers, Maria Sara Mejia de Tafur, Daniel Wallach, Rafael Muñoz-Carpena, Senthold Asseng

**Affiliations:** a International Center for Tropical Agriculture, km 17 recta Cali–Palmira, 763537, Cali, Colombia; b Universidad Nacional UN–Palmira, Colombia; c Department of Agricultural and Biological Engineering, University of Florida, Frazier Rogers Hall, PO Box 110570, Gainesville, FL 32611-0570, USA; d International Institute of Tropical Agriculture, Ibadan, Nigeria; e Institute for Sustainable Food Systems, University of Florida, Gainesville, FL 326110-0570, USA; f CGIAR Research Program on Climate Change, Agriculture and Food Security (CCAFS), c/o CIAT, Cali, Colombia; g INRA, UMR 1248 Agrosystèmes et développement territorial (AGIR), 31326 Castanet-Tolosan Cedex, France

**Keywords:** Enhanced sampling uniformity (eSU), Sobol, DSSAT, Sensitivity analysis

## Abstract

We identified the most sensitive genotype-specific parameters (GSPs) and their contribution to the uncertainty of the MANIHOT simulation model. We applied a global sensitivity and uncertainty analysis (GSUA) of the GSPs to the simulation outputs for the cassava development, growth, and yield in contrasting environments. We compared enhanced Sampling for Uniformity, a qualitative screening method new to crop simulation modeling, and Sobol, a quantitative, variance-based method. About 80% of the GSPs contributed to most of the variation in maximum leaf area index (LAI), yield, and aboveground biomass at harvest. Relative importance of the GSPs varied between warm and cool temperatures but did not differ between rainfed and no water limitation conditions. Interactions between GSPs explained 20% of the variance in simulated outputs. Overall, the most important GSPs were individual node weight, radiation use efficiency, and maximum individual leaf area. Base temperature for leaf development was more important for cool compared to warm temperatures. Parameter uncertainty had a substantial impact on model predictions in MANIHOT simulations, with the uncertainty 2–5 times larger for warm compared to cool temperatures. Identification of important GSPs provides an objective way to determine the processes of a simulation model that are critical versus those that have little relevance.

## Highlights

• Model outputs showed sensitivity to about 80 % of the 16 genetic parameters.
• At least 20 % of the output variance is due to interactions among parameters.
• Importance of parameters varied between warm and cool environments.
• Sensitivity of parameters did not differ between rainfed and unlimited conditions.
• Uncertainty due to crop model parameters can be larger than crop model uncertainty.

## 1 Introduction

Crop models require many input variables and parameters, which can limit their usefulness to represent crop performance (Grassini et al., 2015; Hunt and Boote, 1998; Makowski et al., 2006; Zhang et al., 2016). For example, genotype specific parameters (GSPs) must apply to crops grown under both limiting and non-limiting conditions. Estimating them, therefore, requires observations from experiments for a wide range of environments. The quantity and quality of the data used to estimate the GSPs is important; inaccurate GSPs cause large uncertainty in simulated crop performance (Hoogenboom et al., 2012; Makowski et al., 2006; Muñoz-Carpena et al., 2007). It is, therefore, critical to understand the sensitivity of a model to each GSP and its impact on model uncertainty (Confalonieri et al., 2010c; Esmaeili et al., 2014; Saltelli et al., 2000b; Xing et al., 2017).

A sensitivity analysis of a model evaluates the sensitivity of model outputs based on the variation of the input variables and GSPs (Cariboni et al., 2007; Saltelli et al., 2008a, 2000a; Vazquez-Cruz et al., 2014). A sensitivity analysis can be local or global, depending on whether it evaluates the effect of changing parameters one at a time, or varying all the parameters simultaneously (Confalonieri et al., 2010a; Saltelli et al., 2008a, 2000a). Uncertainty analysis, in contrast, quantifies how variation in input parameters affects model outputs and is used to select the factors that cause most uncertainty in the outputs (Saltelli et al., 2008b, 2004). Sensitivity and uncertainty analysis are often conducted at the same time (Saltelli et al., 2008a).

Cassava (*Manihot esculenta* Crantz) is an important crop for food security and income for small-holder farmers in developing countries. Cassava is resilient to drought and high temperatures and responds positively to an increase in the atmospheric CO_2_ concentration. Cassava may, therefore, become more relevant under climate change (Jarvis et al., 2012; Rosenthal and Ort, 2012). A new simulation model for cassava, MANIHOT, was incorporated in the Decision Support System for Agrotechnology Transfer package (DSSAT;www.DSSAT.net) (Hoogenboom et al., 2019a, 2019b; Jones et al., 2003). MANIHOT is based on the earlier CROPSIM cassava model (Matthews and Hunt, 1994). The changes to the CROPSIM model required new or modified GSPs. The definition and function of each GSP is specified, but we do not know the range each can take for different cultivars nor how it might interact with other GSPs. Moreover, MANIHOT has only been evaluated for a limited number of different regions. We undertook a sensitivity and uncertainty analysis of MANIHOT to identify which parameters contribute the most to model uncertainty.

Sensitivity and uncertainty analyses have been applied extensively in crop modeling. Most studies, however, used uniform distribution to sample the parameter space (e.g. Esmaeili et al., 2014; Makowski et al., 2006; Vazquez-Cruz et al., 2014; White et al., 2005), and rarely sampled from a normal distribution (e.g. Confalonieri et al., 2010b; Dzotsi et al., 2013; Richter et al., 2010). Only one study so far has used distributions of the variables that were analyzed beyond a uniform and normal distribution (Ma et al., 2000). The goal of this study was to determine the nature of the distribution of each GSP and to conduct a global sensitivity and uncertainty analysis (GSUA) based on an enhanced sampling for uniformity (eSU).

## 2 Materials and methods

We conducted the GSUA with the MANIHOT crop simulation model of DSSAT (Hoogenboom et al., 2019a) for four contrasting growing environments using a two-step process. We first defined the probability distribution of each parameter based on a literature review and we then used the eSU method to select parameter values (Chitale et al., 2017; Khare et al., 2015). The eSU is a new qualitative method untested for crop models. Next, we used a quantitative variance-based method (Sobol, 1993) to determine the percentage of variance in the outputs that was explained by each parameter (Saltelli et al., 2009).

### 2.1 The MANIHOT model

MANIHOT was released as a new module for cassava in version 4.7 of DSSAT (Hoogenboom et al., 2019b). It has a simplified branching, treats leaves as cohorts, and new algorithms for the rates of leaf formation and stem growth. It also includes a new water stress factor, a spill-over strategy for biomass allocation, and different cardinal temperatures for branching, leaf growth and leaf age. MANIHOT represents the indeterminate growth and development of cassava, which, unlike many crops, does not have critical phenological phases, nor distinct physiological maturity. Crop development is driven by accumulated thermal time with different cardinal temperatures for the process of branching, and for potential size, age, and growth of leaves. The main changes to the GSPs from the CROPSIM model are:

• The GSPs defining branching time were reduced from 6 to 2;
• The GSPs that define the fraction of assimilate designated for storage root growth (SRFR) and storage root number per unit of canopy weight (SR#W) were removed;
• The modified algorithm for leaf growth reduced the GSPs from 6 to 1; and
• The GSPs for leaf appearance slope (LNSLP), node weight (NODWT) and node length (NODLT) were added.

We refer to the GSPs by the mnemonic used in the DSSAT genetic descriptions for cultivar and ecotype (Table 1). B01ND (time to first branching) establishes the difference between early- and late-branching cultivars. After the first branch has formed, branching continues at a constant rate specified by B12ND. The rate of leaf formation is described by a saturation growth rate, where the interval between the appearance of new nodes increases as the crop ages. MANIHOT uses node as the basic growth unit, which includes the leaf and internode section of stem. The nodal growth rate is represented by a logistic function based on node age and cumulative number of leaves when the node appears. Potential leaf size increases from planting and reaches a maximum value when the cumulative thermal age of the crop is 900-degree days (°Cd). LAXS is the potential area of an individual leaf (Table 1) at 900 °C d and declines as the crop ages. LLIFA is the duration in thermal time after the leaf reaches its full size and before it senesces. A cohort of nodes is a group that are all created at the same time. All new branches and nodes of a cohort are symmetric. The number of nodes in a cohort is determined by the total number of apices, which is defined by the number of branches per fork (BR1F–BR4F, Table 1).

**Table 1:** Input probability distribution function (PDF) for the crop parameters in the MANIHOT cassava model. The PDFs were used to define the sampling for the global sensitivity and uncertainty analysis and were obtained from reported data of each parameter through a literature review.

GSP	Parameter description	PDF^a^	Statistics	K–S^b^	AIC^c^	n^d^	Sources
B01ND	Thermal time from planting to first branching (°Cd)	Triangular^e^	min(a) = 189; max(b) = 1447; mode(c)=764	0.105 (0.97)	313.4	22	i
B12ND	Mean thermal time between branching levels after the first branching (°Cd)	Triangular	min = 284; max = 899; mode=456	0.084 (0.99)	279.9	22	i
LAXS	Maximum individual leaf area (cm^2^)	Lognormal	μY = 5.748; σY = 0.314; truncation= (0.001-0.9)	0.070 (0.93)	725.2	60	j
SLAS	Specific leaf area (cm^2^/g)	Normal	μ = 242.613; σ = 59.975; truncation= (0.1-0.9)	0.217 (0.25)	246.6	22	k
LLIFA	Active leaf area duration in thermal time (°Cd) after full expansion	Weibull	shape(a) = 4.183; scale(b) = 1015.34; left truncation (value, c) = 100; right truncation (probability) = 0.9	0.039 (0.69)	4682.5	338	l
LPEFR	Leaf-petiole weight fraction (-)	Gamma	shape(a) = 8.984; scale(s) = 0.0237; left truncation (value, b) = 0.1; right truncation (probability) = 0.9	0.087 (0.01)	−845.9	337	m
LNSLP	Leaf appearance slope (-) as proportion of the leaf appearance curve of reference f	Uniform	min = 0.7; max = 1.3			0^g^	
NODWT	Individual node weight (g)	Weibull	shape(a) = 3.157; scale(b) = 9.253; left truncation (value, c) = 1; right truncation (probability) = 0.9	0.082 (0.69)	359.9	72	n
NODL	Internode length (cm)	Lognormal	μY = 0.502; σY = 0.398; truncation= (0.001-0.9)	0.090 (0.76)	113.9	55	o
PARUE	Radiation use efficiency (g dry matter MJ^−1^)	Lognormal	μY = 0.337; σY = 0.310; truncation= (0.1-0.9)	0.201 (0.20)	35.6	27	p
TBLSZ	Base temperature for leaf development (˚C)	Uniform	min = 11; max = 17			3^h^	q
BR1F-BR4F	Branch number per fork at fork 1–4 (#)	Uniform	min = 1; max=4			2	r
KCAN	Photosynthetically active radiation (PAR) extinction parameter (-)	Uniform	min = 0.58; max = 1.01	0.219 (0.61)		12	s

a See Appendix B. Probability Density Functions (PDF).

b Numbers in parenthesis are the p-values of the Kolmogorov-Smirnov. Null hypothesis: True distribution function of the data is equal to the hypothesized distribution function.

c Akaike information criterion.

d n: Number of observations.

e Weibull distribution had the best fit followed by the triangular distribution; however triangular distribution was selected because it is already truncated.

f The leaf appearance curve of reference was estimated in thermal time from 4 varieties in 3 locations using data from Irikura et al. (1979).

g The threshold of the parameter LNSLP was defined based on initial attempts of calibration of this parameter for different varieties and locations.

h The uniform distribution was set for the parameters with few observations.

i (Bolaños, 1987; Irikura et al., 1979; Lian and Cock, 1979; Veltkamp, 1986).

j (Alves, 2002; Alves and Setter, 2004, 2000; Ceballos and Cruz, 2012; CIAT, 1978, 1975; Connor et al., 1981; Gabriel et al., 2014; Irikura et al., 1979; Keating et al., 1982; Lian and Cock, 1979; Matthews and Hunt, 1994; Okogbenin et al., 2013; Pellet and El-Sharkawy, 1993; Pinheiro et al., 2014; Santhosh Mithra et al., 2013; Streck et al., 2014; Veltkamp, 1986).

k (Fukai and Hammer, 1987; Gabriel et al., 2014; Gijzen et al., 1990; Keating et al., 1982; Matthews and Hunt, 1994; Pellet and El-Sharkawy, 1993; Rosenthal and Ort, 2012; Vandegeer et al., 2013).

l (Alves, 2002; Bolaños, 1987; CIAT, 1978; Connor et al., 1981; Fukai and Hammer, 1987; Howeler, 2011; Irikura et al., 1979; Lian and Cock, 1979; Veltkamp, 1986).

m (Cadavid, 1988; CIAT, 2013a; Cock, 2011; De Tafur et al., 1994; Fukai and Hammer, 1987).

n (CIAT. Unpublished results., n.d.; CIAT, 2013b; Lian and Cock, 1979; Porto, 1983).

o (CIAT. Unpublished results., n.d.).

p (De Souza et al., 2016; El-Sharkawy and Mejia de Tafur, 2010; Ezui, 2017; Leepipatpaiboon et al., 2009; Pellet and El-Sharkawy, 1997; Veltkamp, 1986; Yamamoto et al., 2004).

q (Fukai and Hammer, 1987; Keating et al., 1982; Manrique, 1992).

r (Ceballos and Cruz, 2012; Lian and Cock, 1979).

s (Cock, 2011; Ezui, 2017; Fukai and Hammer, 1987; June, 1993; Pellet and El-Sharkawy, 1993; Veltkamp, 1986).

Potential crop growth is estimated by summing potential stem and leaf growth plus 10% to account for the growth of fibrous roots. The stem growth rate is calculated by adding the growth rate for all the cohorts, while the leaf growth rate is calculated by summing the potential leaf size for each cohort multiplied by the specific leaf area (SLAS).

MANIHOT uses a spill-over strategy where the daily assimilation is first allocated to satisfy the requirements for growth of aboveground biomass and fibrous roots with only the remainder allocated to growth of storage roots. Storage roots therefore have no fixed initiation. Daily assimilate from photosynthesis in MANIHOT is calculated by multiplying the solar radiation intercepted each day by radiation use efficiency (PARUE). The potential crop growth rate is estimated each day as the total demand for assimilate to satisfy the growth of leaves, stems and fibrous roots. When the daily assimilation is less than the amount required to satisfy the potential growth, the actual growth of nodes and fibrous roots is reduced proportionally. Only when daily assimilation is greater than the demand to satisfy the potential growth is the excess allocated to the storage roots.

MANIHOT (Moreno-Cadena, 2018), includes a new drought stress factor based on soil water content rather than the ratio between potential and actual transpiration as used in several other DSSAT crop models (Ritchie, 1998). The drought stress factor affects the germination, leaf appearance, branching, leaf size and biomass increase. The sensitivity to drought can be different for germination but it is the same for the other processes. Appendix A includes a brief evaluation of the results. The source code is available from GitHub under the 3-Clause BSD License (https://github.com/DSSAT/dssat-csm-os).

### 2.2 Input parameters and their distributions

**Fig. 1:**
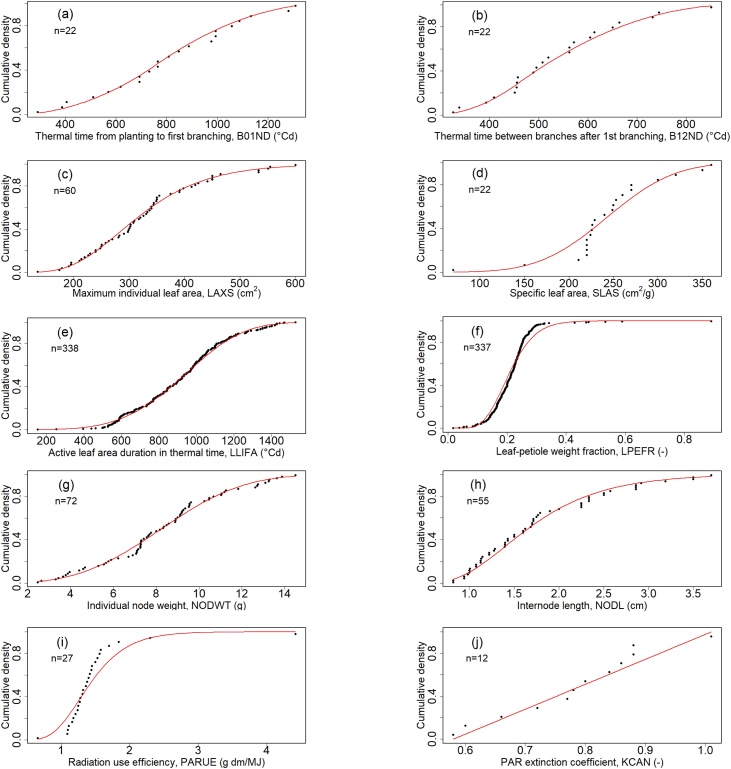
Cumulative distribution functions (red line) of ten GSPs based on literature data (dots) (Table 1). GSPs with less than ten observations were assumed uniform and are not presented. (a) Thermal time from planting to first branching (B01ND); (b) thermal time between branches after first branching (B12ND); (c) maximum individual leaf area (LAXS); (d) specific leaf area (SLAS); (e) active leaf area duration in thermal time (LLIFA), (f) leaf-petiole weight fraction (LPEFR), (g) individual node weight (NODWT), (h) internode length (NODL), (i) radiation use efficiency (PARUE), (j) photosynthetically active radiation (PAR) extinction (KCAN). (For interpretation of the references to colour in the Figure, the reader is referred to the web version of this article).

MANIHOT currently has 18 GSPs. The effects of photoperiod (photoperiod sensitivity, PPS1) and nitrogen (nitrogen concentration in the storage roots, SRN%S) so far have not implemented in the code and were, therefore, omitted from this study. The distribution of the remaining 16 GSPs was defined based on a literature review of 35 publications and an unpublished database of the International Center for Tropical Agriculture (CIAT) (Table 1 and Fig. 1). Where necessary, we converted reported chronological time to thermal time using the mean temperature of the study site.

The distribution of each GSP represents the structure of the variability among cultivars including different timing and patterns of branching, leaf sizes and leaf retention. We fitted different distributions to the data of each GSP using the R *fitdistrplus* package. We selected the distribution that provided the best fit according to the Kolmogorov-Smirnov (K–S) statistic and the Akaike information criterion (AIC) (Delignette-Muller and Dutang, 2015; R Core Team, 2018). We used a uniform distribution for those GSPs that had less than 10 observations to avoid unrealistic assumptions about the distribution (Muñoz-Carpena et al., 2007; Vanuytrecht et al., 2014). MANIHOT uses a reference curve for the rate of leaf appearance based on experimental data for four varieties from three locations (Irikura et al., 1979). The GSP LNSLP is an index that adjusts the slope of the reference curve to provide the observed values of leaf number for the relevant cultivar.

### 2.3 Study sites and model input data

The sensitivity of a simulation model to the variation of a GSP depends on the growth environment (Confalonieri et al., 2010b; Silvestro et al., 2017; Vanuytrecht et al., 2014). We, therefore, conducted the global sensitivity analysis of MANIHOT under combinations of warm and cool temperatures under either rainfed or no water limitations (subsequently termed *unlimited*). We selected two sites in Colombia, Popayan (2.4278 °N, 76.6208 °W, 1750 masl), and Cereté (8.8397 °N, 75.8019 °W, 20 masl) with contrasting temperatures because of their different altitudes. Both are important national cassava areas and represent 27 % and 62 % of the global and equatorial cassava production, respectively (IFPRI and IIASA, 2005; Kottek et al., 2006). The mean temperature for the growing season from late April to February was 18 °C in Popayan (cool), and 28 °C in Cereté (warm). Rainfall and mean solar radiation during the growing season were 890 mm and 16 MJ/m^2^/day for Popayan and 1130 mm and 17 MJ/m^2^/day for Cereté (Table 2).

**Table 2:** Description of the study sites used for the sensitivity and uncertainty analysis. The temperature, solar radiation and rainfall are mean values during the growing season for the study period. The temperature classifications reflect the 10 °C difference between the two sites due to altitude.

Site	Temperature classification	Latitude (°)	Longitude (°)	Altitude (masl)	Mean temperature (°C)	Mean solar radiation (MJ/m^2^/day)	Mean rainfall (mm)
Popayan	Cool	2.4278	−76.6208	1750	18	16	890
Cereté	Warm	8.8397	−75.8019	20	28	17	1130

We obtained 30 years’ data of maximum and minimum temperature, precipitation and solar radiation for each site (Popayan, 1984–2013, Cereté, 1980–2009). The weather data for Cereté were obtained from IDEAM, the Colombian National Institute of Meteorology; the weather data for Popayan were obtained from NASA/POWER (Stackhouse et al., 2018; Van Wart et al., 2015). We quality checked the data (Esquivel et al., 2018) with the RClimTool software (Llanos-Herrera, 2014). The soil data for Cereté were from the IRI database (IRI et al., 2015) and for Popayan the site’s profile data in the DSSAT database (see Table C1, Table C2 in Appendix C).

### 2.4 Global sensitivity analysis by enhanced sampling Uniformity (eSU)

We used eSU to rank GSPs according their relative influence on MANIHOT’s outputs of the simulated processes. We selected eSU because it creates multiple combinations of GSPs with improved sample uniformity, sample spread and screening efficiency compared with other methods (Chitale et al., 2017; Khare et al., 2015). We used the eSU procedure in the MATLAB package (The MathWorks Inc, 2015) to create a set of 408 combinations within 8 levels of each of the 16 GSPs (k) specifying 24 trajectories (r). Trajectories are defined as the succession of points within the sampling space that create the r*(k+1) combinations (Chitale et al., 2017). We specified 8 levels of each GSP, which is more than 4 that is commonly used for these analyses (Chitale et al., 2017; Khare et al., 2015; Saltelli et al., 2008b). The number of trajectories specified must be a multiple of the number of levels and typically ranges 10–30 (Chitale et al., 2017).

We ran MANIHOT using each of the 408 GSPs combinations for each location for 30 years. We selected six output variables: aboveground biomass, yield, maximum leaf area index (LAI), number of leaves at harvest, and time to appearance of the first and the second branch and calculated their 30-year means. We used sensitivity analysis to identify the first- and high-order effects of the individual GSPs. First-order or elementary effects (μ) are a direct influence of a GSP in an output. High-order effects of GSPs on an output are either interactions between one GSPs and all the others or the non-linear effects of a particular GSP (Saltelli et al., 2004; Wallach et al., 2014). We used a modified version of the elementary effects (μ*) (Campolongo et al., 2007) and standard deviation (σ) for the high interactions or non-linear effects of the GSPs on the output variables.

We normalized values of the elementary effects (μ*) for each output variable by dividing them by the maximum value of the elementary effects of all GSPs across the four treatments. We similarly normalized the values of the standard deviations (σ) for each output by dividing by the maximum value of the σ for all GSPs.

### 2.5 Global uncertainty and sensitivity analysis using the Sobol methodology

We selected as important the GSPs with normalized μ* or σ greater than 0.5 for at least one output variable for any of the two locations following Loubière et al. (2016) and Chitale et al. (2017). We used the Sobol method to evaluate the uncertainty of the model outputs due to the uncertainty of the selected GSPs. Again, we used the mean value of the output variables over the 30 years of simulations to estimate the cumulative probability function for the selected output variables. We also estimated the 95% confidence intervals due to the weather variation over the 30 years.

Sobol decomposes output variability into the contributions of the individual GSPs and their corresponding interactions, termed first and total order sensitivity indices, respectively. The first sensitivity index of a given GSP is the proportion of the total variance of the output variable explained by its main effect (Giglioli and Saltelli, 2003). The total sensitivity index of a GSP includes the first order sensitivity index plus the interactions of the GSP with all others.

We used the same probability distribution functions as in the eSU analysis to create the Monte Carlo samples for the Sobol method (Table 1 and Fig. 1). The Sobol analysis required 15,360 GSP combinations, given by 2n(k+1), where k is the number of GSPs and n (>500) is the number of samples to estimate the individual effects. We specified 14 GSPs (k) and a sample size of 512 (n) in SimLab v2.2.1 (Khare et al., 2015). We used the data for the same six output variables as in the eSU analysis. To evaluate the similarities between the two methods for each GSP, we compared the elementary effects from eSU with the total order indices from Sobol.

### 2.6 Crop model simulations

The simulations for both sites started 30 days prior to planting with the initial soil water content set to field capacity. We did not simulate additional limitations due to nutrients, weeds or pests.

## 3 Results

### 3.1 Distribution of reported data of GSPs

The number of observations in the literature of the GSPs differed widely (Table 1). We found more than 330 observations for the active leaf area duration (LLIFA) and leaf-petiole weight fraction (LPEFR). In contrast, we found few data for the base temperature for leaf development (TBLSZ) and for the number of branches per fork (BR1F-BR4F).

Either normal, lognormal, gamma, Weibull, uniform or triangular distributions fitted the data of those GSPs for which we found enough data for the fitting routine to converge (Fig. 1). The fits met the K–S test at a confidence level P < 0.05 for all the GSPs except for LPEFR, which indicates that the functions describe their true distribution. Although the gamma function gave an acceptable fit to the observed data of LPEFR (Fig. 1(f)) the K–S test rejected the null hypothesis.

### 3.2 Global sensitivity analysis: Screening method using the enhanced Sampling Uniformity (eSU)

**Fig. 2:**
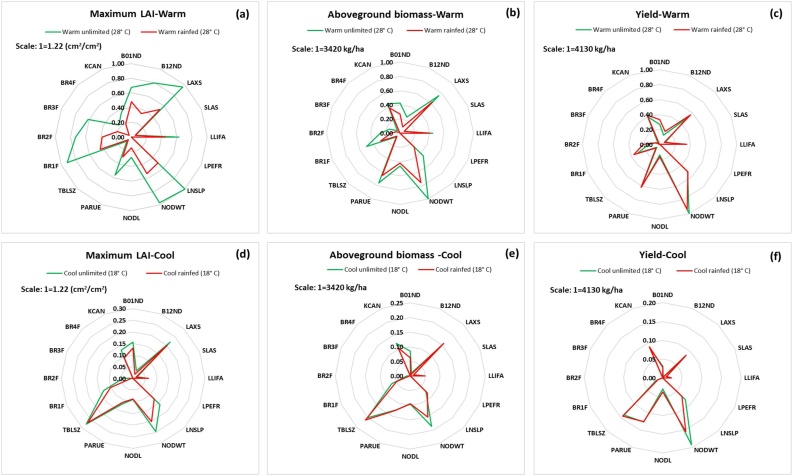
Normalized elementary effects (μ*) in the output variables of MANIHOT due to the variation of each of the GSPs using the enhanced Sampling for Uniformity method of maximum leaf area index (LAI) **(a, d)**, aboveground biomass **(b, e)**, and yield **(c, f)** at warm (top) and cool (bottom) environments under unlimited (green) and rainfed (red) conditions. Note that the maximum values of μ* are shown at the left top corner of each column. The minimum value was 0 overall. The definitions for the GSPs are listed in Table 1.

For all six output variables there was no difference in sensitivity to the GSPs between rainfed and unlimited conditions (Fig. 2), in contrast to the warm and cold environments. The base temperature for leaf development (TBLSZ) is more important under low temperatures. Values of both μ* and σ were higher for the warm environment because thermal time accumulates faster, which increases the values of the simulated outputs. The most important GSPs were individual node weight (NODWT), maximum individual leaf size (LAXS), thermal time from planting to first branching (B01ND) and PAR extinction coefficient (KCAN). Most GSPs affected the maximum LAI (Fig. 2 a, d) with 10 of the 16 values of μ* values above 0.5 in the warm environment with water unlimited. In contrast, only seven of them had μ* values above 0.15 in the cool environment. As expected, branching behavior simulated by MANIHOT is sensitive to the GSPs B01ND and B12ND (data not shown).

**Fig. 3:**
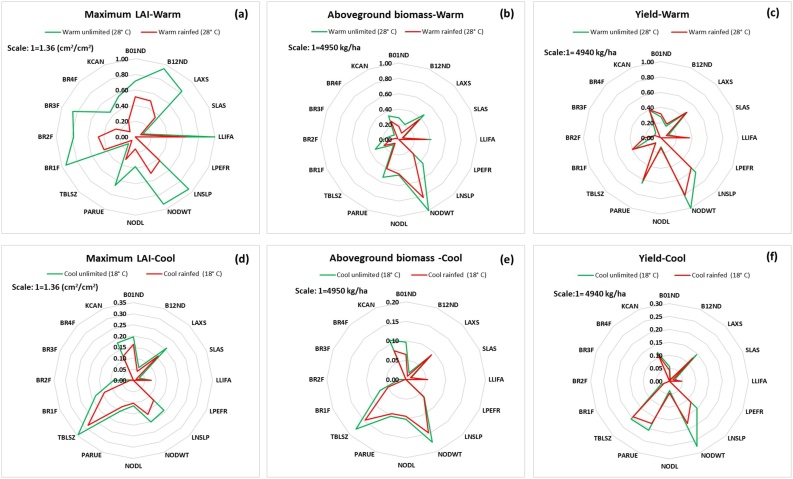
Normalized standard deviation of elementary effects (σ) in the output variables of MANIHOT due to the variation of each of the GSPs using the enhanced Sampling for Uniformity method of maximum leaf area index (LAI) **(a, d)**, aboveground biomass **(b, e)**, and yield **(c, f)** at warm (top) and cool (bottom) environments under unlimited (green) and rainfed (red) conditions. Note that the maximum values of σ are shown at the left top corner of each column. The minimum value was 0 overall. The definitions for the GSPs are listed in Table 1.

Like μ*, the values of σ under rainfed and unlimited conditions were similar, but differed between warm and cool environments (Fig. 3). Most of the GSPs that had high direct effects (μ*) (Fig. 2) also had high interactions (σ). For warm temperatures, active leaf area duration (LLIFA) and number of branches at the third branching level (BR3F) showed a larger interaction than direct effects. Radiation use efficiency (PARUE) also had larger values of σ than of μ*.

As expected, both PARUE and LAXS are key GSPs that increase LAI, aboveground biomass and yield. High values for the base temperature for leaf development (TBLSZ) decreased maximum LAI, aboveground biomass and yield. The effect of node weight (NODWT) on aboveground biomass depended on its magnitude (termed *non-monotonic*). Increasing values up to a threshold increased aboveground biomass while values higher than the threshold also affected partitioning, because it also reduced the assimilate available for leaf growth.

### 3.3 Uncertainty analysis: Sobol method

**Fig. 4:**
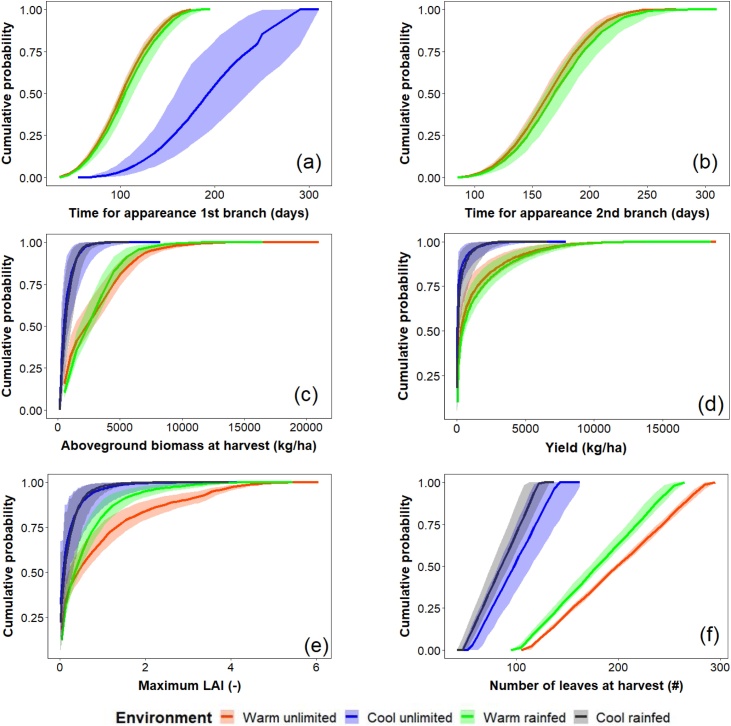
Cumulative probability for the output variables of MANIHOT obtained from the Sobol method at cool and warm environments rainfed and under unlimited conditions: **(a)** Time for appearance of first branch; **(b)** Time for appearance of second branch, **(c)** aboveground biomass at harvest (kg/ha); **(d)** Yield (kg/ha); **(e)** Maximum LAI; and **(f)** Number of leaves at harvest. The solid lines represent the cumulative probability of the 30-year means of the simulated values. The shaded areas are the cumulative probabilities of the 95 % confidence intervals.

As expected, the Sobol method provided a wide range of values for each GSP, which caused the simulation outputs of MANIHOT to vary widely. All six outputs (Fig. 4) for both warmer environments covered wider ranges than those for the cool environment except for the time to appearance of the first branch (Fig. 4a). Under cool growing conditions, uncertainty in the MANIHOT outputs due to uncertainty in the input GSPs was similar for both rainfed and water-unlimited conditions (Fig. 4). For the warm environment, maximum LAI and number of leaves at harvest with unlimited water were higher than rainfed, which increased aboveground biomass but reduced yield slightly.

The time to branching had a coefficient of variation (CV) of 30% and 20% in the time to first and second branching, respectively (Table 3). The aboveground biomass had a CV between 74% and 88% due to parameter uncertainty, while the maximum LAI and yield had a higher CV with values above 120%.

**Table 3:** Standard deviation and coefficient of variation of six output variables of MANIHOT obtained from the global uncertainty analysis of the input GSPs using the Sobol method in the four test environments (see text). Estimates of the standard deviation used the mean values of the 30 years of simulations for each environment.

Variable	Standard deviation				Coefficient of variation (CV, %)			
	warm unlimited	warm rainfed	cool unlimited	cool rainfed	warm unlimited	warm rainfed	cool unlimited	cool rainfed
Time for appearance 1 st branch (days)	30	31	51		29	30	26	
Time for appearance 2nd branch (days)	33	35			20	20		
Aboveground biomass at harvest (kg/ha)	2498	1981	598	556	86	74	88	76
Yield (kg/ha)	2037	2152	481	452	163	149	227	177
Maximum LAI (-)	1.163	0.688	0.332	0.273	125	117	159	129
Number of leaves at harvest (#)	51	45	25	21	25	25	25	25

### 3.4 Global sensitivity analysis: Sobol method

**Fig. 5:**
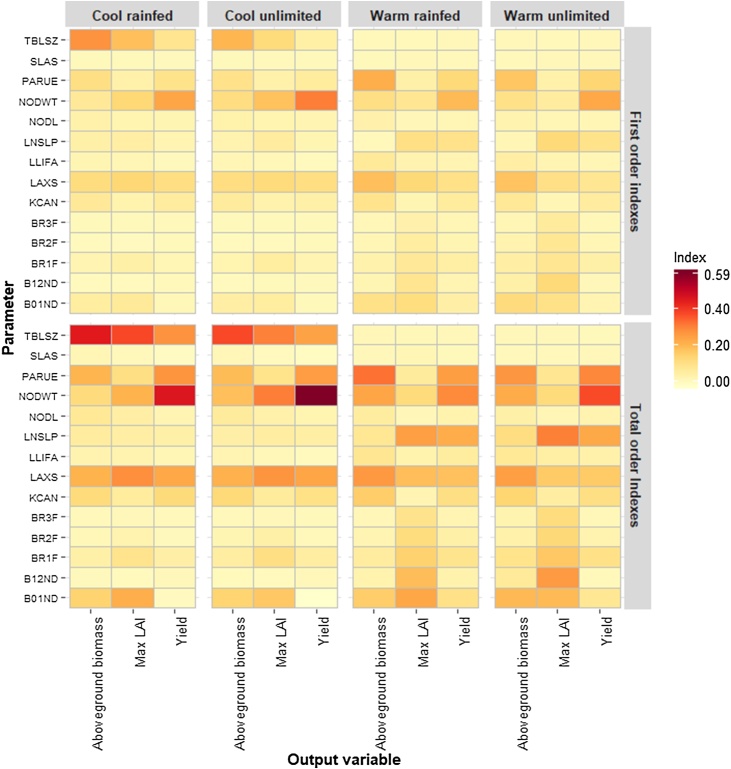
Proportion of variance of aboveground biomass, maximum LAI and yield explained by each GSP in the Sobol analysis. The first order sensitivity index (main effect) (top) and total order sensitivity index (main effect plus interactions) (bottom) for cool and warm temperatures under rainfed and water-unlimited conditions.

Individual node weight (NODWT), radiation use efficiency (PARUE) and maximum individual leaf size (LAXS) were the most important GSPs (Fig. 5) based on their direct effect or first order indices. Temperature modified the importance of some GSPs. Base temperature for leaf development (TBLSZ) accounted for the variation for the cool compared to the warmer environments. Leaf appearance slope (LNSLP) had higher direct effect under warm temperatures than under low temperatures.

The direct effect of individual GSPs explained 55–80% of the variance of the simulated output variables, depending on the temperature and water regime. Individual node weight (NODWT) contributed most to total uncertainty for yield for the cool and unlimited environment, explaining 30% of the variance. The base temperature for leaf development (TBLSZ) was the next most important GSP, explaining 27% of the variance for aboveground biomass for the same environment (Fig. 5).

Interactions between GSPs account for 20–45% of the variance in the output variables. Both eSU and Sobol showed that GSPs with high direct effects also had high interactions (eSU) or high total order indices (Sobol). Sobol also agrees with eSU in attributing the variance of simulated maximum LAI to the influence of many GSPs and their interactions. Sobol showed that the GSP specific leaf area (SLAS) had low first and total order sensitivity for all simulated output variables for all four environments.

**Fig. 6:**
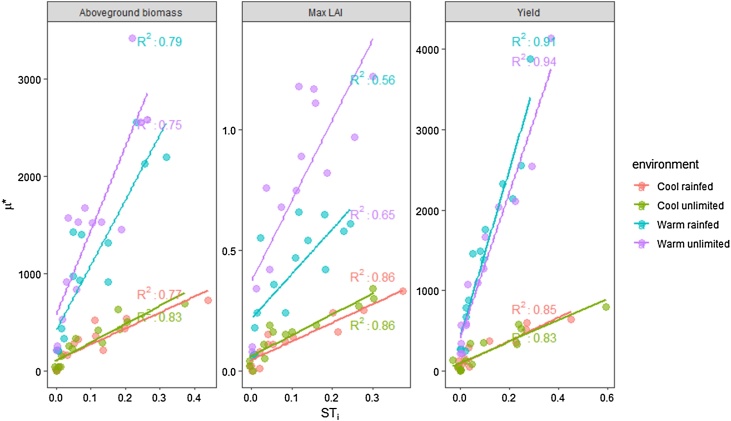
Elementary effects from eSU (μ*) versus the total order indices of Sobol (ST_i_) for cool and warm temperatures under rainfed and water-unlimited conditions. Note that the units of μ* are those of the simulated output variables.

There was a strong positive linear relationship between the total order sensitivity index (main effect plus interactions between parameters, ST_i_ in Sobol) and the elementary effects of each parameter (eSU) (Fig. 6). The relation was similar for all the output variables with the highest R^2^ for yield. The slopes of the relationship differ according to the temperature of each environment.

## 4 Discussion

This study presents a new comprehensive approach to sensitivity and uncertainty analysis of a crop model for contrasting environments. We defined the probability distributions for the range of uncertainty of each GSP based on experimental measurements from a thorough literature search. Previous studies on the sensitivity and uncertainty analysis for crop modeling commonly used uniform distribution of input parameters based on limited references (e.g. Esmaeili et al., 2014; He et al., 2016; Makowski et al., 2006; Silvestro et al., 2017; Vazquez-Cruz et al., 2014; Xing et al., 2017). Knowing the actual distribution of the input parameters (GSPs) allowed us to specify a more realistic representation of the results of the sensitivity and uncertainty analysis. In contrast, an inadequate representation of the range of the parameters inevitably degrades the quality of the sensitivity analysis (Wang et al., 2013; Zhao et al., 2014).

Plasticity of a simulation model is its tendency to change its sensitivity to the GSPs under different environments (e.g. Silvestro et al., 2017). The MANIHOT simulation model showed low plasticity (little variation) in the relevance of the GSPs between rainfed and water unlimited conditions. Both the WOFOST model for wheat (He et al., 2016) and CSM-CROPGRO-Cotton model (Pathak et al., 2007) showed similar plasticity in the response to the water regime. In contrast, the Aquacrop model for wheat (Xing et al., 2017) and the SALUS model for maize (Dzotsi et al., 2013) are more sensitive to some GSPs than to others when the crop is rainfed.

Temperature had a significant effect on the plasticity of MANIHOT. The GSP base temperature for leaf development (TBLSZ) is much more important at low temperatures. The maximum value for TBLSZ is close to the mean temperature of the cool environment (18 °C). Thermal time for leaf growth therefore accumulates only slowly, resulting in a low LAI and hence less interception of solar radiation and thus a smaller production of assimilates. The result is less aboveground biomass and low yield. The SALUS model for maize, peanut and cotton showed a similar sensitivity to the GSPs for different locations (Dzotsi et al., 2013), although the ranking changed. The base temperature used to estimate photothermal time was therefore more important for cooler climates, which is similar to MANIHOT. Sensitivity analyses conducted with other crop models also found that the base temperature is commonly identified as an important GSP. Examples include the STAMINA model for wheat (Richter et al., 2010), the WOFOST model for maize (Wang et al., 2013), the GROSUB model for rye (Feyereisen et al., 2006), and the CropSyst and WARM models for rice (Confalonieri et al., 2006).

Other studies have shown plasticity of their GSPs under different environmental conditions for the Aquacrop model for maize (Vanuytrecht et al., 2014) and the APSIM model for wheat (Zhao et al., 2014). The different conditions included varying temperature and rainfall distribution.

Plasticity allows a model to reflect performance of a cultivar in different environments, which may require more GSPs. The disadvantage is the cost to estimate the extra GSPs, especially if there are many cultivars with different phenotypes (Silvestro et al., 2017).

As discussed above, cassava grows slower under low temperatures. This is reflected in lower values of μ* and σ for all the GSPs, and is a common characteristic of crop models (e.g. Silvestro et al. (2017) for wheat for both the Aquacrop and SAFYE models).

Overall, the most important GSPs were the individual node weight (NODWT), radiation use efficiency (PARUE), and maximum individual leaf area (LAXS). The base temperature for leaf development (TBLSZ) only became important at low temperatures. Radiation use efficiency is commonly one of the most important GSPs because it controls the carbohydrates available for daily growth (e.g. Confalonieri et al., 2010a, 2010b, 2006; Dzotsi et al., 2013; Feyereisen et al., 2006; Makowski et al., 2006; Wang et al., 2013). This version of MANIHOT does not include the detailed stomatal response of VPD but it has a GSP for the day-to-day effect of VPD on photosynthesis, which likely modifies the sensitivity of PARUE. It was not included in the sensitivity analysis because it is a species-wide parameter.

Sensitivity analyses for short-season annual crops has shown that crop yield is very sensitive to GSPs associated with accumulation of thermal time and phenological development (He et al., 2015; Zhao et al., 2014). However, the GSPs in MANIHOT that define branching time, which are the analogs of phenological stages in other crops, accounted for less than 20 % of the variance in yield.

Neither leaf-petiole weight fraction (LPEFR) nor the fourth branching level (BR4F) affected the simulation outputs of MANIHOT. The sensitivity analysis used over 300 values for LPEFR, but they varied little from 0.2. We suggest that LPERF is redundant as a GSP and can be replaced with a constant as part of the species description. Under cool temperatures, the MANIHOT model rarely simulates to four branches within 10 months so that the GSP for the fourth branching level (BR4F) has much less impact in the analyses. Nevertheless, under warm temperatures the fourth branching level (BR4F) interacts with GSPs that control leaf size and thus affects the plant’s leaf area.

The simulation outputs maximum LAI, aboveground biomass and yield, showed low sensitivity to the GSP specific leaf area (SLAS), which we did not expect. SLAS does control leaf growth through its direct effect on LAI. Nevertheless, aboveground biomass is dominated by stem weight so that the effect of SLAS alone may not be detectable.

The simulated outputs were sensitive to about 80% of the GSPs through direct effects and 20% through interactions. This emphasizes that it is important to account for the interactions when estimating the values of GSPs during model calibration. GSPs with higher interactions may require evaluation of multiple combinations of GSPs.

The variability for simulated variables in MANIHOT due to parameter uncertainty was larger than for crop simulation models such as wheat in which the coefficient of variation for yield is about 13% (Asseng et al., 2013). The uncertainty of simulation outputs in MANIHOT was larger for warm and rainfed conditions than in either water regime in cool environments, in which temperature was the main limiting factor. In contrast, for a warm temperature environment, water-unlimited simulations produced higher values of maximum leaf area index, number of leaves and aboveground biomass. The water-unlimited regime for warm temperatures gave different cumulative probabilities and higher CVs for the simulated outputs compared with rainfed conditions.

MANIHOT is based on the spill-over concept in which assimilate is allocated to satisfy demand to fulfil potential growth of aboveground organs. Only assimilate surplus above these demands are allocated to the storage roots. Under warm temperatures, water-unlimited conditions, therefore, resulted in a yield that was slightly lower than under rainfed conditions because proportionally more of the available assimilate was allocated to the aboveground organs, leaving less surplus. This contrasts with cereal crops where the harvested grain is an integral aboveground organ and well-watered crops produce a higher yield compared to rainfed (DeJonge et al., 2012).

The elementary effects from eSU and the total order indices from Sobol were highly correlated. This shows the utility of eSU to screen GSPs for models with many input parameters when Sobol could challenge marginal computational infrastructure. Similar findings of high correlations between qualitative and quantitative methods have been reported (e.g. Confalonieri et al., 2010a; Stella et al., 2014). However, this study is the first instance where the qualitative eSU procedure has been used for analysis of a crop simulation model.

Sensitivities to GSPs values in MANIHOT differed between growing environments giving a large range of model uncertainty. GSPs must, therefore, be estimated across contrasting growing environments to ensure reliable model simulations. We only considered the uncertainty of the GSPs but the same procedures could be applied to other input variables that contribute to model uncertainty such as soil characteristics (DeJonge et al., 2012; Jones et al., 2012).

## 5 Conclusions

The enhanced Sampling for Uniformity (eSU), showed that maximum LAI, yield, and aboveground biomass at harvest simulated by MANIHOT were sensitive to about 80% of the genotype specific parameters (GSPs). The importance of GSPs in the simulated variables did not change between water-unlimited and rainfed conditions but differed between warm and cool environments. The most important GSPs were individual node weight, radiation use efficiency and maximum individual leaf area. The base temperature for leaf development was more relevant at cool than at warm temperatures. About 20% of the variance in the output variables was due to GSP interactions, which is important when estimating parameter values. Further research should include sensitivity and uncertainty analysis of other input variables such as soil characteristics. The comprehensive global sensitivity analysis approaches that we developed here can readily be applied to other crop models. It also provides an objective way to identify processes included in simulation models that have little importance.

## CRediT authorship contribution statement

**Leidy Patricia Moreno-Cadena:** Conceptualization, Methodology, Validation, Formal analysis, Writing - original draft. **Gerrit Hoogenboom:** Conceptualization, Methodology, Resources, Writing - review & editing, Supervision. **Myles James Fisher:** Writing - review & editing. **Julian Ramirez-Villegas:** Writing - review & editing. **Steven Dean Prager:** Writing - review & editing. **Luis Augusto Becerra Lopez-Lavalle:** Project administration, Supervision. **Pieter Pypers:** Funding acquisition, Project administration. **Maria Sara Mejia de Tafur:** Conceptualization. **Daniel Wallach:** Methodology, Writing - original draft. **Rafael Muñoz-Carpena:** Conceptualization, Methodology. **Senthold Asseng:** Conceptualization, Methodology, Writing - review & editing, Supervision.

## Declaration of Competing Interest

None.

## Figures and Tables

**Fig. A1:**
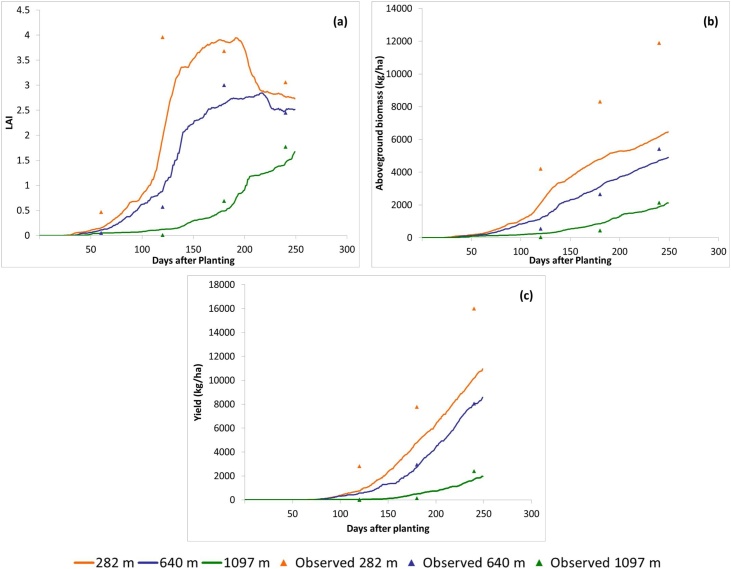
Observed (triangles) and simulated (lines) values using the MANIHOT model of leaf area index (LAI) **(a)** aboveground biomass (kg/ha) **(b)** and yield (kg/ha) **(c)** for the variety Ceiba grown at 282 m (orange), 640 m (blue) and 1097 m (green) on the Island of Maui in Hawaii. The experimental data were extracted from Manrique (1992) (reproduced from Moreno-Cadena (2018)).

**Fig. A2:**
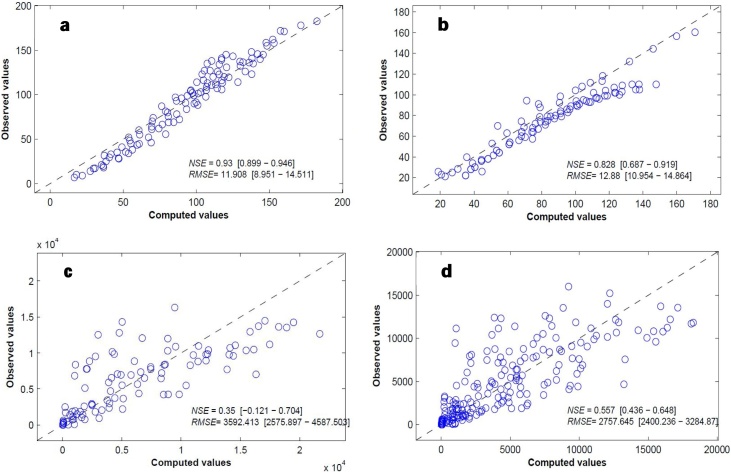
Scatterplot of observed versus computed values in the calibration (left) and evaluation (right) of the MANIHOT model for the variables leaf number **(a, b)** and yield (kg/ha) **(c, d)**. Each plot registers the Nash & Sutcliffe efficiency coefficient (NSE) and the root mean square error (RMSE) (reproduced from Moreno-Cadena (2018)).

**Table C1:** Physical and chemical soil properties of the experiment in Popayan.

ID	Color	Albedo	Evaporation limit (mm)	Drainage coefficient	Runoff curve	Photosynthesis factor^1^				
CCBN880060	Black	0.09	8.7	0.6	76	1				
Soil depth (cm)	Lower limit (cm^3^ cm^−3^)	Drained upper limit (cm^3^ cm^−3^)	Saturation (cm^3^ cm^−3^)	Soil root growth factor	Saturated hydraulic conductivity (cm h^−1^)	Bulk density (g cm^−3^)	Organic carbon (%)	Clay (%)	Silt (%)	pH (water)
5	0.075	0.215	0.406	0.5	−99	0.58	16.8	9	61.6	5.7
13	0.075	0.215	0.406	0.5	−99	0.58	16.8	9	61.6	5.7
30	0.039	0.175	0.371	0.2	−99	0.55	15.8	0.9	53	4.9
39	0.05	0.164	0.302	0.1	−99	0.52	6.87	0	18.5	5.1
66	0.043	0.139	0.302	0.1	−99	0.48	3.8	0	14	5.3
82	0.041	0.13	0.302	0.1	−99	0.51	2.19	0	12.4	5.3
102	0.044	0.14	0.302	0.1	−99	0.62	1.88	0	14.2	5.3
124	0.04	0.128	0.302	0.1	−99	0.64	1.51	0	12	5.2
137	0.045	0.145	0.302	0	−99	0.51	1.66	0	15.2	5.2
176	0.037	0.117	0.302	0	−99	0.48	1.31	0	10	5.2
200	0.033	0.104	0.302	0	−99	0.4	1.65	0	7.7	5.6

1 The photosynthesis factor was set to 1 although the original value in the database of DSSAT is 0.9.

**Table C2:** Physical and chemical soil properties of the experiment in Cereté.

ID	Color	Albedo	Evaporation limit (mm)	Drainage coefficient	Runoff curve	Photosynthesis factor				
CO04204610	Black	0.1	6	0.5	75	1				
Soil depth (cm)	Lower limit (cm^3^ cm^−3^)	Drained upper limit (cm^3^ cm^−3^)	Saturation (cm^3^ cm^−3^)	Soil root growth factor	Saturated hydraulic conductivity (cm h-1)	Bulk density (g cm^−3^)	Organic carbon (%)	Clay (%)	Silt (%)	pH (water)
5	0.154	0.265	0.399	1	0.62	1.42	2.01	25.62	25.31	5.78
15	0.166	0.278	0.403	0.85	0.49	1.44	1.7	27.68	24.5	5.85
30	0.182	0.295	0.408	0.7	0.37	1.47	1.3	30.26	23.33	5.94
60	0.197	0.312	0.414	0.5	0.27	1.52	0.83	32.78	22.11	6.06
100	0.196	0.31	0.413	0.38	0.28	1.58	0.48	32.72	21.54	6.2
200	0.186	0.298	0.408	0.05	0.34	1.63	0.28	31.06	21.33	6.37

## Appendix A Evaluation of the MANIHOT model

This Appendix is based on the model calibration and evaluation presented in the MSc thesis of Moreno-Cadena (2018).

The performance of the cassava model at different temperatures is shown on Fig. A1. The simulations are based on the experiment reported by Manrique (1992) at 282 m, 640 m and 1097 m with mean temperatures of 22.4 °C, 20.6 °C and 18.1 °C, respectively. While the data at 640 m were used for calibration, the other two locations were used to evaluate the model. The weather data were generated using the monthly mean values reported in the publication using WeatherMan. The soil data for all the locations were from the IRI database. The model captures the differences in leaf area index (LAI) and biomass due to different temperatures.

Moreno-Cadena (2018) compared MANIHOT simulations with data from ten experiments with nine varieties at three sites in Colombia. The model represented leaf number with a root mean square error (RMSE) less than 13 for both the calibration and evaluation data sets, tending to underestimate at the end of the growing season. Simulated yield of total dry matter was more variable (RMSE up to 3600 kg/ha and a coefficient of determination (R^2^) of 0.62) (Fig. A2). Although both GUMCAS (Matthews and Hunt, 1994) and LINTUL (Ezui et al., 2018) reported higher R^2^ values (0.76 and 0.92), they were for just one variety at one (GUMCAS) or two (LINTUL) sites.

## Appendix B Probability Density Functions (PDF)

(B.1) $$\text{T}\text{r}\text{i}\text{a}\text{n}\text{g}\text{u}\text{l}\text{a}\text{r}:\frac{2\left(\text{x}-\text{a}\right)}{\left(\text{b}-\text{a}\right)\left(\text{c}-\text{a}\right)}\;\text{f}\text{o}\text{r}\;\text{a}\leq \text{x}\leq \text{c},\frac{2\left(\text{b}-\text{x}\right)}{\left(\text{b}-\text{a}\right)\left(\text{b}-\text{c}\right)}\;\text{f}\text{o}\text{r}\;\text{c}<\text{x}\leq \text{b}$$

(B.2) $$\text{L}\text{o}\text{g}\;\text{n}\text{o}\text{r}\text{m}\text{a}\text{l}:\frac{1}{\left(\sigma Yx\sqrt{2\;\pi }\;\right)}\text{exp}\left(-\left(\frac{{\left(\ln\left(\;x\right)\;–\;\mu Y\right)}^{2}}{2\;{\left(\sigma Y\right)}^{2}}\right)\right)$$

(B.3) $$\text{N}\text{o}\text{r}\text{m}\text{a}\text{l}:\frac{1}{\sigma \sqrt{2\;\pi }\;}\text{exp}\left(-\left(\frac{{\left(x-\;\mu \right)}^{2}}{2\;\sigma^{2}}\right)\right)$$

(B.4) Weibull:abx-cba-1exp-x-cba

(B.5) $$\text{G}\text{a}\text{m}\text{m}\text{a}:\;\left(\frac{1}{\left(s^{a}\;\Gamma \left(a\right)\right)}\right){\left(x-b\right)}^{a-1}\text{exp}\left(-\left(\frac{x-b}{s}\right)\right)\text{w}\text{h}\text{e}\text{r}\text{e}\;\text{Γ}\;\text{i}\text{s}\;\text{t}\text{h}\text{e}\;\text{g}\text{a}\text{m}\text{m}\text{a}\;\text{f}\text{u}\text{n}\text{c}\text{t}\text{i}\text{o}\text{n}\;$$

(B.6) $$\text{U}\text{n}\text{i}\text{f}\text{o}\text{r}\text{m}\;\to \;\frac{1}{max-min}$$

## References

[bib0005] Alves A.A.C., Hillocks R., Thresh J., Bellotti A.C. (2002). Cassava botany and physiology. Cassava: Biology, Production and Utilization.

[bib0010] Alves A.A.C., Setter T.L. (2000). Response of cassava to water deficit. Crop Sci..

[bib0015] Alves A.A.C., Setter T.I.M.L. (2004). Response of cassava leaf area expansion to water deficit: cell proliferation, cell expansion and delayed development. Ann. Bot..

[bib0020] Asseng S., Ewert F., Rosenzweig C., Jones J.W., Hatfield J.L., Ruane A.C., Boote K.J., Thorburn P.J., Rötter R.P., Cammarano D., Brisson N., Basso B., Martre P., Aggarwal P.K., Angulo C., Bertuzzi P., Biernath C., Challinor A., Doltra J., Gayler S., Goldberg R., Grant R., Heng L., Hooker J., Hunt L.A., Ingwersen J., Izaurralde R.C., Kersebaum K.C., Müller C., Naresh Kumar S., Nendel C., O’Leary G., Olesen J.E., Osborne T.M., Palosuo T., Priesack E., Ripoche D., Semenov M.A., Shcherbak I., Steduto P., Stöckle C., Stratonovitch P., Streck T., Supit I., Tao F., Travasso M., Waha K., Wallach D., White J.W., Williams J.R., Wolf J. (2013). Uncertainty in simulating wheat yields under climate change. Nat. Clim. Chang..

[bib0025] Bolaños A.C. (1987). Análisis De Crecimiento Para Tres Formas De Propagacion En Yuca. Tesis Biólogo-botánico.

[bib0030] Cadavid L.F. (1988). . Efecto de fertilización y humedad relativa sobre la absorción y distribución de nutrimentos en yuca. Tesis De Maestría, Facultad De Ciencias Agropecuarias..

[bib0035] Campolongo F., Cariboni J., Saltelli A. (2007). An effective screening design for sensitivity analysis of large models. Environ. Model. Softw..

[bib0040] Cariboni J., Gatelli D., Liska R., Saltelli A. (2007). The role of sensitivity analysis in ecological modelling. Ecol. Modell..

[bib0045] Ceballos H., Cruz G., Ospina B., Ceballos H. (2012). Cassava Taxonomytaxonomy and morphology. Cassava in the Third Millennium: Modern Production, Processing, Use, and Marketing Systems.

[bib0050] Chitale J., Khare Y., Muñoz-Carpena R., Dulikravich G.S., Martinez C. (2017). An effective parameter screening strategy for high dimensional models. ASME Int. Mech. Eng. Congr. Expo..

[bib0055] CIAT (1975). CIAT Annual Report.

[bib0060] CIAT (1978). Cassava Annual Report.

[bib0065] CIAT (2013). Institutional Memory: Crecimiento Del Cultivo De La Yuca En Tres Formas De Propagación.

[bib0070] CIAT (2013). Institutional Memory: Efecto Del Rocío Sobre El Desarrollo De Mcol-22.

[bib0075] CIAT. Unpublished results, n.d. Ensayos Convenio Ministerio de Agricultura de Colombia y CIAT: Clima y sector agropecuario colombiano, adaptación para la sostenibilidad productiva.

[bib0080] Cock J.H., Howeler R. (2011). Cassava growth and development. Ed.), The Cassava Handbook..

[bib0085] Confalonieri R., Acutis M., Bellocchi G., Cerrani I., Tarantola S., Donatelli M., Genovese G. (2006). Exploratory sensitivity analysis of CropSyst, WARM and WOFOST: a case study with rice biomass simulations. Ital. J. Agrometeorol..

[bib0090] Confalonieri R., Bellocchi G., Bregaglio S., Donatelli M., Acutis M. (2010). Comparison of sensitivity analysis techniques: a case study with the rice model WARM. Ecol. Modell..

[bib0095] Confalonieri R., Bellocchi G., Tarantola S., Acutis M., Donatelli M., Genovese G. (2010). Sensitivity analysis of the rice model WARM in Europe: exploring the effects of different locations, climates and methods of analysis on model sensitivity to crop parameters. Environ. Model. Softw..

[bib0100] Confalonieri R., Bregaglio S., Acutis M. (2010). A proposal of an indicator for quantifying model robustness based on the relationship between variability of errors and of explored conditions. Ecol. Modell..

[bib0105] Connor D.J., Cock J.H., Parra G.E. (1981). Response of cassava to water shortage I. Growth and yield. F. Crop. Res..

[bib0110] Core Team R. (2018). R: a Language and Environment for Statistical Computing.

[bib0115] De Souza A.P., Massenburg L.N., Jaiswal D., Cheng S., Shekar R., Long S.P. (2016). Rooting for cassava: insights into photosynthesis and associated physiology as a route to improve yield potential. New Phytol..

[bib0120] De Tafur S.M., Cadavid Lopez L.F., El-Sharkawy M.A. (1994). Respuesta de la yuca (*Manihot esculenta* Crantz) al déficit de agua y fertilización. Suelos Ecuatoriales.

[bib0125] DeJonge K.C., Ascough J.C., Ahmadi M., Andales A.A., Arabi M. (2012). Global sensitivity and uncertainty analysis of a dynamic agroecosystem model under different irrigation treatments. Ecol. Modell..

[bib0130] Delignette-Muller M., Dutang C. (2015). Fitdistrplus: an R package for fitting distributions. J. Stat. Softw..

[bib0135] Dzotsi K.A., Basso B., Jones J.W. (2013). Development, uncertainty and sensitivity analysis of the simple SALUS crop model in DSSAT. Ecol. Modell..

[bib0140] El-Sharkawy M.A., Mejia de Tafur S. (2010). Comparative photosynthesis, growth, productivity, and nutrient use efficiency among tall- and short-stemmed rain-fed cassava cultivars. Photosynthetica.

[bib0145] Esmaeili S., Thomson N.R., Tolson B.A., Zebarth B.J., Kuchta S.H., Neilsen D. (2014). Quantitative global sensitivity analysis of the RZWQM to warrant a robust and effective calibration. J. Hydrol. (Amst).

[bib0150] Esquivel A., Llanos-Herrera L., Agudelo D., Prager S.D., Fernandes K., Rojas A., Valencia J.J., Ramirez-Villegas J. (2018). Predictability of seasonal precipitation across major crop growing areas in Colombia. Clim. Serv..

[bib0155] Ezui K.S. (2017). Understanding the Productivity of Cassava in West Africa.

[bib0160] Ezui K.S., Leffelaar P.A., Franke A.C., Mando A., Giller K.E. (2018). Simulating drought impact and mitigation in cassava using the LINTUL model. F. Crop. Res..

[bib0165] Feyereisen G.W., Sands G.R., Wilson B.N., Strock J.S., Porter P.M. (2006). Plant growth component of a simple rye growth model. Trans. ASABE.

[bib0170] Fukai S., Hammer G.L. (1987). A simulation model of the growth of the cassava crop and its use to estimate cassava productivity in northern Australia. Agric. Syst..

[bib0175] Gabriel L.F., Streck N.A., Roberti D.R., Chielle Z.G., Uhlmann L.O., da Silva M.R., da Silva S.D. (2014). Simulating cassava growth and yield under potential conditions in Southern Brazil. Agron. J..

[bib0180] Giglioli N., Saltelli A. (2003). Simlab 2.2, Software for sensitivity and uncertainty analysis, Simlab manual. Jt. Res. Cent. Eur. Comm.

[bib0185] Gijzen H., Veltkamp H.J., Goudriaan J., Bruijn G.Hde (1990). Simulation of dry matter production and distribution in cassava (*Manihot esculenta* Crantz). Netherlands J. Agric. Sci..

[bib0190] Grassini P., van Bussel L.G.J., Van Wart J., Wolf J., Claessens L., Yang H., Boogaard H., de Groot H., van Ittersum M.K., Cassman K.G. (2015). How good is good enough? Data requirements for reliable crop yield simulations and yield-gap analysis. F. Crop. Res..

[bib0195] He L., Zhao G., Jin N., Zhuang W., Yu Q. (2015). Global sensitivity analysis of APSIM-Wheat parameters in different climate zones and yield levels. Trans. Chinese Soc. Agric. Eng..

[bib0200] He L., Hou Y., Zhao G., Wu D., Yu Q. (2016). Parameters optimization of WOFOST model by integration of global sensitivity analysis and Bayesian calibration method. Trans. Chinese Soc. Agric. Eng..

[bib0205] Hoogenboom G., Jones J.W., Traore P.C.S., Boote K.J., Kihara J., Fatondji D., Jones W.J., Hoogenboom G., Tabo R., Bationo A. (2012). Experiments and data for model evaluation and application. Improving Soil Fertility Recommendations in Africa Using the Decision Support System for Agrotechnology Transfer (DSSAT.

[bib0210] Hoogenboom G., Porter C.H., Boote K.J., Shelia V., Wilkens P.W., Singh U., White J.W., Asseng S., Lizaso J.I., Moreno L.P., Pavan W., Ogoshi R., Hunt L.A., Tsuji G.Y., Jones J.W., Boote K.J. (2019). The DSSAT crop modeling ecosystem. Advances in Crop Modeling for a Sustainable Agriculture..

[bib0215] Hoogenboom G., Porter C.H., Shelia V., Boote K.J., Singh U., White J.W., Hunt L.A., Ogoshi R., Lizaso J.I., Koo J., Asseng S., Singels A., Moreno L.P., Jones J.W. (2019). Decision Support System for Agrotechnology Transfer (DSSAT).

[bib0220] Howeler R. (2011). The Cassava Handbook: a Reference Manual Based on the Asian Regional Cassava Training Course, Held in Thailand.

[bib0225] Hunt L.A., Boote K.J., Tsuji G.Y., Hoogenboom G., Thornton P.K. (1998). Data for model operation, calibration, and evaluation. Understanding Options for Agricultural Production.

[bib0230] IFPRI, IIASA (2005). Global Spatially-disaggregated Crop Production Statistics Data for 2005 Version 3.2.

[bib0235] IRI, MSU, HarvestChoice, IFPRI (2015). Global High-resolution Soil Profile Database for Crop Modeling Applications.

[bib0240] Irikura Y., Cock J.H., Kawano K. (1979). The physiological basis of genotype—temperature interactions in cassava. F. Crop. Res..

[bib0245] Jarvis A., Ramirez-Villegas J., Herrera Campo B.V., Navarro-Racines C. (2012). Is cassava the answer to African climate change adaptation?. Trop. Plant Biol..

[bib0250] Jones J., Hoogenboom G., Porter C., Boote K.J., Batchelor W.D., Hunt L.A., Wilkens P.W., Singh U., Gijsman A.J., Ritchie J.T. (2003). The DSSAT cropping system model. Eur. J. Agron..

[bib0255] Jones J., Naab J., Fatondji D., Dzotsi K., Adiku S., He J., Kihara J., Fatondji D., Jones J.W., Hoogenboom G., Tabo R., Bationo A. (2012). Uncertainties in simulating crop performance in degraded soils and low input production systems. Improving Soil Fertility Recommendations in Africa Using the Decision Support System for Agrotechnology Transfer (DSSAT).

[bib0260] June T. (1993). The effect of light on growth of cassava and sorghum I light distribution and extinction coefficient. J. Agromet Indones..

[bib0265] Keating B.A., Evenson J.P., Fukai S. (1982). Environmental effects on growth and development of cassava (*Manihot esculenta* Crantz.) I. Crop development. F. Crop. Res..

[bib0270] Khare Y.P., Muñoz-Carpena R., Rooney R.W., Martinez C.J. (2015). A multi-criteria trajectory-based parameter sampling strategy for the screening method of elementary effects. Environ. Model. Softw..

[bib0275] Kottek M., Grieser J., Beck C., Rudolf B., Rubel F. (2006). World map of the Köppen-Geiger climate classification updated. Meteorol. Zeitschrift.

[bib0280] Leepipatpaiboon S., Boonyawat S., Sarobol E. (2009). Estimation of solar radiation use efficiency in paddy and cassava fields. Kasetsart J. Soc. Sci..

[bib0285] Lian T.S., Cock J.H. (1979). Cassava plant forms and their associated morpho-physiological characters. MARDI Res Bull.

[bib0290] Llanos-Herrera L. (2014). RClimTool.

[bib0295] Loubière P., Jourdan A., Siarry P., Chelouah R. (2016). A sensitivity analysis method for driving the Artificial Bee Colony algorithm’s search process. Appl. Soft Comput..

[bib0300] Ma L.C., Ascough J.R., Ahuja L.J., Shaffer M.D., Hanson J.W., Rojas K. (2000). Root zone water quality model sensitivity analysis using Monte Carlo simulation. Trans. ASAE.

[bib0305] Makowski D., Naud C., Jeuffroy M.-H., Barbottin A., Monod H. (2006). Global sensitivity analysis for calculating the contribution of genetic parameters to the variance of crop model prediction. Reliab. Eng. Syst. Saf..

[bib0310] Manrique L.A. (1992). Growth and yield performance of cassava grown at three elevations in Hawaii. Commun. Soil Sci. Plant Anal..

[bib0315] Matthews R.B., Hunt L. (1994). GUMCAS: a model describing the growth of cassava (*Manihot esculenta* Crantz). F. Crop. Res..

[bib0320] Moreno-Cadena L.P. (2018). Modelo De Simulación De Yuca (Manihot Esculenta Crantz) En El Trópico. Magister En Ciencias Agrarias Con Énfasis En Fisiología De Cultivos.

[bib0325] Muñoz-Carpena R., Zajac Z., Kuo Y.M. (2007). Global sensitivity and uncertainty analyses of the water quality model VFSMOD-W. Trans. Asabe.

[bib0330] Okogbenin E., Setter T., Ferguson M., Mutegi R., Ceballos H., Olasanmi B., Fregene M. (2013). Phenotypic approaches to drought in cassava: review. Front. Physiol..

[bib0335] Pathak T.B., Fraisse C.W., Jones J., Messina C.D., Hoogenboom G. (2007). Use of global sensitivity analysis for CROPGRO cotton model development. Trans. ASABE.

[bib0340] Pellet D., El-Sharkawy M.A. (1993). Cassava varietal response to phosphorus fertilization. II. Phosphorus uptake and use efficiency. F. Crop. Res..

[bib0345] Pellet D.M., El-Sharkawy M.A. (1997). Cassava varietal response to fertilization: growth dynamics and implications for cropping sustainability. Exp. Agric..

[bib0350] Pinheiro D.G., Streck N.A., Richter G.L., Langner J.A., Winck J.E.M., Uhlmann L.O., Zanon A.J. (2014). Limite crítico no solo para transpiração e crescimento foliar em mandioca em dois períodos com deficiência hídrica. Rev. Bras. Ciência do Solo.

[bib0355] Porto M. (1983). Physiological Mechanisms of Drought Tolerance in Cassava (*Manihot Esculenta* Crantz).

[bib0360] Richter G.M., Acutis M., Trevisiol P., Latiri K., Confalonieri R. (2010). Sensitivity analysis for a complex crop model applied to Durum wheat in the Mediterranean. Eur. J. Agron..

[bib0365] Ritchie J.T., Tsuji G.Y., Hoogenboom G., Thornton P.K. (1998). Soil water balance and plant water stress. Understanding Options for Agricultural Production.

[bib0370] Rosenthal D.M., Ort D. (2012). Examining cassava’s potential to enhance food security under climate change. Trop. Plant Biol..

[bib0375] Saltelli A., Chan K., Scott M. (2000). Sensitivity Analysis, Probability and Statistics Series.

[bib0380] Saltelli A., Tarantola S., Campolongo F. (2000). Sensitivity analysis as an ingredient of modeling. Stat. Sci..

[bib0385] Saltelli A., Tarantola S., Campolongo F., Ratto M., Saltelli A., Tarantola S., Campolongo F., Ratto M. (2004). Methods based on decomposing the variance of the output. Sensitivity Analysis in Practice.

[bib0390] Saltelli A., Ratto M., Andres T., Campolongo F., Cariboni J., Gatelli D., Saisana M., Tarantola S., Saltelli A., Ratto M., Andres T., Campolongo F., Cariboni J., Gatelli D.M.S., Tarantola S. (2008). Sensitivity analysis: from theory to practice. Global Sensitivity Analysis..

[bib0395] Saltelli A., Ratto M., Andres T., Campolongo F., Cariboni J., Gatelli D., Saisana M., Tarantola S., Saltelli A., Ratto M., Andres T., Campolongo F., Cariboni J., Gatelli D., Saisana M., Tarantola S. (2008). Introduction to sensitivity analysis. Eds.), Global Sensitivity Analysis..

[bib0400] Saltelli A., Campolongo F., Cariboni J. (2009). Screening important inputs in models with strong interaction properties. Reliab. Eng. Syst. Saf..

[bib0405] Santhosh Mithra V., Sreekumar J., Ravindran C.S. (2013). Computer simulation of cassava growth: a tool for realizing the potential yield. Arch. Agron. Soil Sci..

[bib0410] Silvestro P.C., Pignatti S., Yang H., Yang G., Pascucci S., Castaldi F., Casa R. (2017). Sensitivity analysis of the Aquacrop and SAFYE crop models for the assessment of water limited winter wheat yield in regional scale applications. PLoS One.

[bib0415] Sobol I.M. (1993). Sensitivity estimates for nonlinear mathematical models. Math. Model. Comput. Exp..

[bib0420] Stackhouse P.W., Zhang T., Westberg D., Barnett A.J., Bristow T., Macpherson B., Hoell J.M. (2018). NASA Prediction of Worldwide Energy Resources, Release 8. arxiv:/URLhttps://power.larc.nasa.gov.

[bib0425] Stella T., Frasso N., Negrini G., Bregaglio S., Cappelli G., Acutis M., Confalonieri R. (2014). Model simplification and development via reuse, sensitivity analysis and composition: a case study in crop modelling. Environ. Model. Softw..

[bib0430] Streck N.A., Pinheiro D.G., Junior Zanon A., Gabriel L.F., Rocha T.S.M., Souza A.Tde, Silva M.Rda (2014). Efeito do espaçamento de plantio no crescimento, desenvolvimento e produtividade da mandioca em ambiente subtropical. Bragantia.

[bib0435] The MathWorks Inc, 2015. MATLAB Version 8.5 (R2015a).

[bib0440] Van Wart J., Grassini P., Yang H., Claessens L., Jarvis A., Cassman K.G. (2015). Creating long-term weather data from thin air for crop simulation modeling. Agric. For. Meteorol.

[bib0445] Vandegeer R., Miller R.E., Bain M., Gleadow R.M., Cavagnaro T.R. (2013). Drought adversely affects tuber development and nutritional quality of the staple crop cassava (Manihot esculenta Crantz). Funct. Plant Biol..

[bib0450] Vanuytrecht E., Raes D., Willems P. (2014). Global sensitivity analysis of yield output from the water productivity model. Environ. Model. Softw..

[bib0455] Vazquez-Cruz M.A., Guzman-Cruz R., Lopez-Cruz I.L., Cornejo-Perez O., Torres-Pacheco I., Guevara-Gonzalez R.G. (2014). Global sensitivity analysis by means of EFAST and Sobol’ methods and calibration of reduced state-variable TOMGRO model using genetic algorithms. Comput. Electron. Agric..

[bib0460] Veltkamp H.J. (1986). Physiological Causes of Yield Variation in Cassava (Manihot Esculenta Crantz).

[bib0465] Wallach D., Makowski D., Jones J.W., Brun F. (2014). Uncertainty and Sensitivity Analysis, in: Working With Dynamic Crop Models.

[bib0470] Wang J., Li X., Lu L., Fang F. (2013). Parameter sensitivity analysis of crop growth models based on the extended Fourier Amplitude Sensitivity Test method. Environ. Model. Softw..

[bib0475] White J.W., Hoogenboom G., Hunt L.A. (2005). A structured procedure for assessing how crop models respond to temperature. Agron. J..

[bib0480] Xing H., Xu X., Li Z., Chen Y., Feng H., Yang G., Chen Z. (2017). Global sensitivity analysis of the AquaCrop model for winter wheat under different water treatments based on the extended Fourier amplitude sensitivity test. J. Integr. Agric..

[bib0485] Yamamoto T., Nawata E., Watanabe K., Sributta A., Noichana C., Sakuratani T. (2004). Simple models of cassava attainable yield estimation for regional-level productivity analysis in Northeast Thailand. Japanese J. Trop. Agric..

[bib0490] Zhang K., Ma J., Zhu G., Ma T., Han T., Feng L.L. (2016). Parameter sensitivity analysis and optimization for a satellite-based evapotranspiration model across multiple sites using Moderate Resolution Imaging Spectroradiometer and flux data. J. Geophys. Res. Atmos..

[bib0495] Zhao G., Bryan B.A., Song X. (2014). Sensitivity and uncertainty analysis of the APSIM-wheat model: interactions between cultivar, environmental, and management parameters. Ecol. Modell..

